# Impact of the implementation of carbon emission trading on corporate financial performance: Evidence from listed companies in China

**DOI:** 10.1371/journal.pone.0253460

**Published:** 2021-07-01

**Authors:** Meijuan Liu, Chang Zhou, Feifei Lu, Xiaohan Hu

**Affiliations:** 1 College of Economics and Management, Zhejiang Agriculture and Forestry University, Hangzhou, China; 2 School of Accounting, Zhejiang University of Finance and Economics, Hangzhou, China; 3 College of Management, Qingdao Agriculture University, Chengyang District, Qingdao, China; 4 Faculty of Agriculture and Forestry, University of Helsinki, Helsinki, Finland; Institute for Advanced Sustainability Studies, GERMANY

## Abstract

With the development of ecological paradigm coupled with the relentless implementation of myriad environmental policies in China, the rapid development of carbon emission trading and carbon trading market has had a vital impact on the financial performance of enterprises at the microlevel. This study has sampled the A-share listed companies in China, from 2009 to 2018, and adopted the difference-in-difference (DID) method to investigate the effect of the carbon emission trading on corporate financial performance from the microlevel. Evidence showed that the implementation of carbon emission trading effectively improved the total asset-liability ratio of enterprises, though it reduced the value of the current capital market. Moreover, in the regions under strict legal environment, the enhancement effect of the total asset-liability ratio was more obvious, whereas in the regions under loose legal environment, the reduction effect of the value of the capital market was more obvious. Further analysis showed that the implementation of carbon emission trading could not promote Chinese enterprises to increase R&D investment. Hence the implementation of carbon emission trading has improved the level of non-business income of enterprises incorporated into the trading system, but its impact on the investment income of enterprises was not significant.

## 1. Introduction

**Fig 1:**
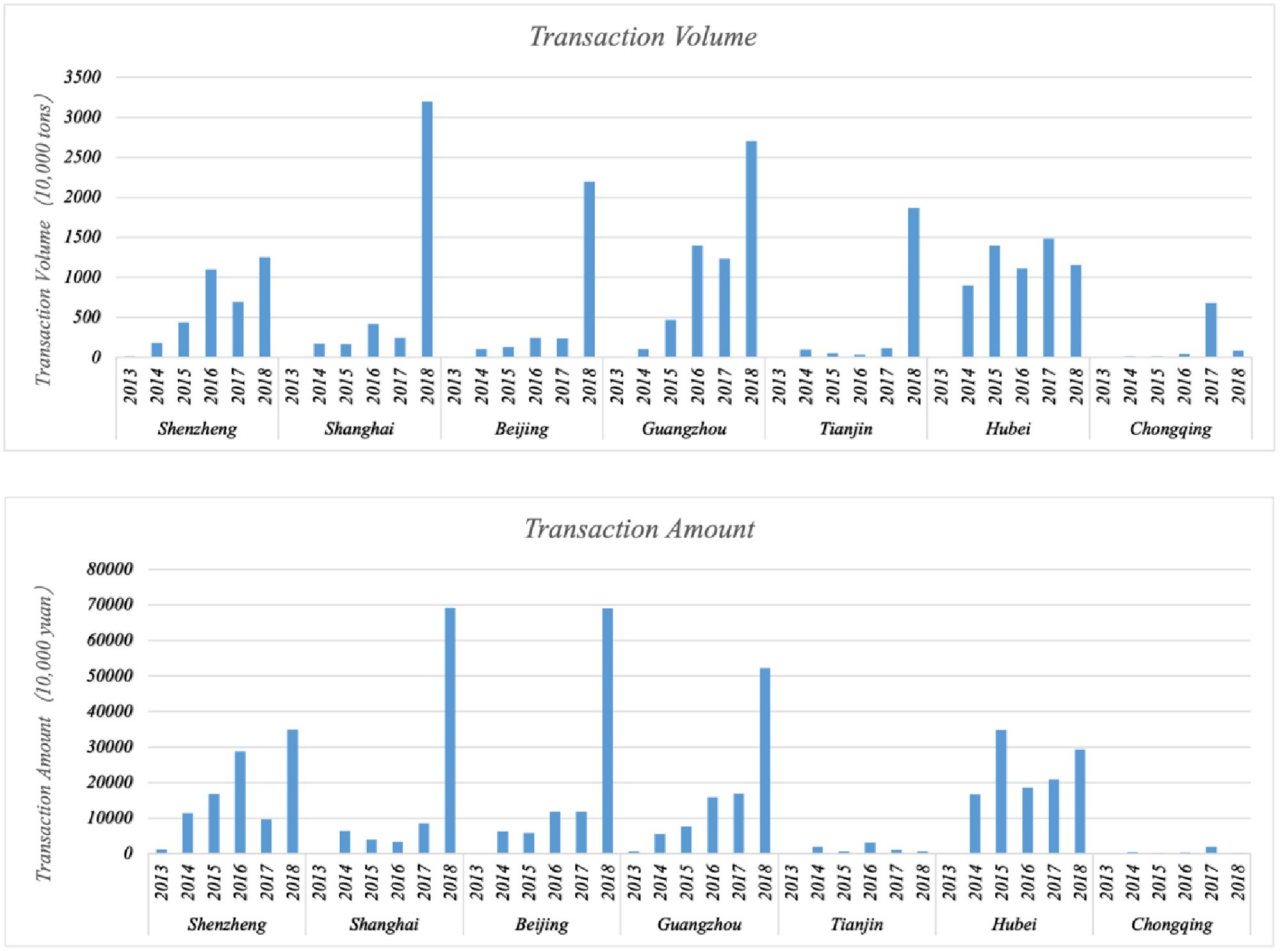
Transaction volume and transaction amount in China’s pilot carbon emission trading market from 2013–2018.

The control of greenhouse gas emission is an important issue figuring out in the current development of all countries across the globe. At the Paris Climate Summit in December 2015, the major countries in the world joined *the Paris Agreement* to formulate “Actions against Climate Change after 2020”. Drawing on the experience of the carbon emission trading markets of the European Union, United States and other countries [1], China began to pilot a carbon emission trading project. The construction of unified carbon emission trading market system in China had been officially launched in 2018. With the rapid development of this pilot market (see Fig 1. Transaction volume and transaction amounts in China’s pilot carbon emission trading market from 2013–2018), the volume and intensity of carbon dioxide emission in the pilot regions have come under control [2–4], and the practice of carbon emission trading has also had a significant impact on the production and operation decisions of enterprises [5,6]. However, the post-policy effects are yet to be fully tested, for example, the impact, owing to the practice of carbon emission trading, on the financial performance of an enterprise, the confirmation of the Porter Hypothesis [7], and the outcome of the “win-win effect” in terms of environmental regulations and the enterprise economy [8].

The government drives the enterprises to internalize the environmental costs through various environmental regulations and policies [9], among which the carbon emission trading mechanism based on Coase’s Property Rights Theory [10] is one of the important measure. However, owing to the difficulty in obtaining data (Countries usually do not require enterprises to disclose or report the detailed information on their carbon emission trading), the current literature does not include the abundant research on the economic outcomes of micro-subjects succeeding the implementation of carbon emission trading. Certain scholars believe that such a practice will increase the total, production, and inventory costs of enterprises [11], increase the R&D expenditures of enterprises owing to the necessity of technological updating [12], or cause depreciation in the corporate value [13,14]. Other scholars have found that the implementation of Clean Development Mechanism (CDM) projects will increase the stock return rate of the company [15], and carbon emission trading can result in the improvement of industrial output value [16,17], corporate value [18], or financial performance [16, 19]. After the launch of the pilot carbon emission trading market by China in 2013, certain Chinese scholars have adopted the DID method to study the relationship between carbon emission reduction and macro-economy [20–22] and proposed that the carbon emission trading can increase the short-term rather than the long-term value of an enterprise. It can be seen that the existing literature has not yet reached an ultimate conclusion on the micro-effects of carbon emission trading on enterprises, and most of the existing studies have used the EU carbon market transaction data or the CDM projects data of China in the past. The economic outcomes of carbon emission trading need further examination over the data from listed Chinese companies.

Based on the above considerations, this study selected 2009–2018 A-share listed Chinese companies as the samples, analyzed the impact of the practice of carbon emission trading on corporate value with the DID method, and further verified the impact of carbon emission trading on the corporate financial performance and R&D investment in the Porter Hypothesis scenario. Evidence showed that the practice of carbon emission trading can effectively increase the total asset-liability ratio of a company, while concomitantly reducing its value in the capital market. In regions with strict legal environments, the effect of increasing the total asset-liability ratio was more obvious whereas in regions with loose legal control, the value reduction in the capital market was more obvious. Further research showed that, whereas the practice of carbon emission trading had not yet promoted the investment of Chinese companies in R&D, it increased the non-operating income of the companies, which have been included in the trading system. However, its impact over corporate return on investment (ROI) was not significant enough.

The main contributions of this work are as listed in the following. First, it makes an empirical analysis on the impact of the carbon emission trading on corporate financial performance, and enriches the research on the economic outcomes of carbon emission trading. Its conclusions provide a theoretical basis for the enterprises to enhance their own value by participating in carbon trading, which demonstrates that the listed companies can improve their corporate environmental management and corporate financial performance by actively participating in the environmental rights trading businesses such as quota trading, Chinese Certified Emission Reduction (CCER) trading, and carbon finance. Second, it has enriched the literature about testing the Porter Hypothesis in the Chinese context. By analyzing the application of the Porter Hypothesis to the carbon emission trading system in China, it proved that the R&D conditions required for the application of the Porter Hypothesis were not yet valid, despite the participation of the Chinese companies in carbon emission trading. Henceforth, it is inevitable for the government and carbon trading institutions to further improve the prevalent market mechanism.

## 2. Theoretical analysis and research hypothesis

### 2.1 The impact of carbon emission trading on the return on corporate assets

Research on the impact of the environmental regulations on corporate value has a long history. Currently, there are mainly three views, as the Traditional, the Porter, and the Uncertainty Hypotheses. Among them, the Traditional Hypothesis holds that the assumption of the enterprise of the environmental responsibility will inevitably lead to the loss of their economic benefits, which weakens their competitiveness to a certain extent, thus inhibiting the improvement of corporate performance. However, an alternate view is that appropriate environmental policies can improve the return on innovation and production efficiency, make up for the costs of the environmental protection of the enterprise and even generate profits [8]. These researchers believe that the environmental regulation can provide a company with innovative and first-mover advantages, and further improve the overall corporate performance [23]. This view has been later developed into the famous Porter Hypothesis and verified by several scholars in the field of economics [24–26]. Furthermore, the Uncertainty Hypothesis argues that there are numerous uncertainties among the environmental regulation and corporate performance.

Based on Property Rights Theory of Coase, Dales [9] proposed an Emission-Trading Program in the field of pollution control. Emission rights refer to the limited rights to use environmental resources. Through government-guided rules, quotas of the pollutant emission rights have been allocated to micro-level emission entities (i.e., enterprises) [27]. Through emission rights transactions between these entities, additional economic output has been created and their total emissions are reduced [28,29]. Accordingly, the market mechanism can play a positive role in the allocation of environmental resources. Furthermore, the environmental subjects are encouraged to make behavioral decisions based on market signals [17,30], so that the marginal costs of pollution reduction will be equal among the emitters, and the total amount of pollutant emissions will be controlled at the lowest overall cost, ultimately achieving Pareto Optimality and sustainable development [4,31].

The practice of carbon emission trading can reduce the emission reduction costs of enterprises [32]. Zubi et al. [33] have proposed that the interregional carbon trading market can produce cost-saving effects, and the cost savings are enhanced if more enterprises are involved in the transactions. Furthermore, carbon emission trading encourages enterprises to make profits by reducing the emissions through market transactions. When the carbon price in the trading market is high, enterprises can have surplus emission quotas through the carbon emission reduction and then sell these extra quotas in the carbon trading market to obtain excess returns. This mechanism is more effective than the controlling-by-commanding tools. Therefore, for the emission entities at the micro level, after practicing the pilot carbon emission trading, additional economic benefits can be obtained through investment activities such as the reduction of costs and generation of trading incomes, thus improving the corporate financial performance.

Based on these studies, the study proposes the following hypothesis:

**Hypothesis 1 (H1a).** *Carbon emission trading can improve a company’s return on assets (ROA)*.
**Hypothesis 2 (H1b).** *Carbon emission trading can reduce a company’s return on assets (ROA)*.

### 2.2 The impact of carbon emission trading on the market value of corporate

Previous studies have drawn diametrically opposite conclusions from the impact of the carbon emission trading on the enhancement of the capital market value of a participant [34]. since quotas of carbon emissions are freely allocated, the selling of the remaining quotas would increase the corporate value [35], thus imposing comparatively loose environmental constraints on enterprises. Because the majority of companies involved in carbon emission trading have been engaged in the energy industry, they can shift the carbon price onto their product prices, bringing them significantly positive yet plenteous returns on their stock prices [36]. From the perspective of the stock value of a company, when the price of the carbon quota increased, the stock price of the company would also increase significantly. Another group of scholars who disagreed with the above views, and argued that carbon emission trading restricted the carbon emission of a company as well as increased the costs of emission reduction, compliance, and technology updates. Furthermore, corresponding incomprehensive disclosure has reduced the market value of the corporate capital [37,38]. Brouwers et al. [39] found that following the EU’s carbon verification, the capital market made a significantly negative response to the companies whose carbon emissions exceeded their quotas.

Currently, the overall transaction amount and volume in the Chinese carbon market have been steadily increasing, and the building of a unified national carbon trading market is in its preliminary stage. Therefore, the allocation of carbon quotas in each pilot market will become increasingly strict. Companies are under the administrative pressure of energy conservation and emission reduction regulations, and have the incentive to purchase the emission reduction equipment and improve the process flow. All this cost information will be exposed to the capital market via corporate financial reports and is likely to reduce the corporate values. Based on the Porter Hypothesis, the current listed Chinese companies disclose little information about their carbon trading. A more detailed disclosure will immediately attract the attention of investors. Energy-saving and emission-reducing companies can gain a “first mover” advantage in the capital market, transmit favorable signals, and have a positive impact on the market value of corporate.

Therefore, this study proposes the following hypothesis:

**Hypothesis 2 (H2a).** *Carbon emission trading can enhance the market value of corporate in the capital market*.
**Hypothesis 2 (H2b).** *Carbon emission trading will reduce the market value of corporate in the capital market*.

### 2.3 The regulatory effect of the legal environment

The legal environment and other institutional factors are important external governance mechanisms for the companies [40,41]. The companies will not take the initiative to reduce emissions for the sake of economy, particularly in terms of the carbon emission reduction [42]. The government must formulate corresponding laws on the regulation and supervision to enable the carbon emission trading. Owing to the imbalance in the economic development among different regions of China, the level of the legal development and the law enforcement environment vastly vary among regions. Henceforth an imbalanced pattern in which the eastern region is better off than the middle region and likewise, the middle region faring better than the western region, has gradually come into existence.

The differences in the legal environment ensure direct benefits that the companies can obtain from participating in the carbon emission trading. In regions where the laws are more complete with a better implementation, companies, whose purchasing and selling of carbon quotas are well protected by the system, can acquire high ROAs. Alternatively, the law as a “visible hand” has a check-and-balance relationship with the capital market. Henceforth, in areas where government regulation is weak, the capital market can play a stronger role instead. Therefore, this study proposes Hypothesis 3:

**Hypothesis 3 (H3a).** *In regions with a strict legal environment*, *the impact of carbon emission trading on corporate ROAs is more significant*.
**Hypothesis 3 (H3b).** *In regions where the legal control is relatively loose*, *carbon emission trading has a more significant impact on corporate values in the capital market*.

## 3. Data and method

### 3.1 Data resources

This study has selected certain Chinese A-share listed companies from 2009 to 2018 as data samples for comparing the impact of enterprise value on the emission control between the regulated and nonregulated enterprises, participating in the carbon trading, before and after the implementation of carbon emission trading. Owing to the different establishment times of carbon emission trading markets in various provinces and cities, the annual selection of samples is different. Therefore, the opening time of pilot markets in each province and city, where the listed companies are located, is selected as the measurement standard. The specific list of enterprises was obtained from the Carbon Emission Trading Enterprises List published by the National Development and Reform Commission of each region, from 2013 to 2018. The paired samples have been selected from enterprises in non-pilot provinces and cities according to the above said variables. Thereafter, the following screening method has been applied: the exclusion of (a) the financial sector, (b) companies that went public after 2010, (c) delisted, ST and *ST companies, and (d) companies with missing data. The data employed in this study have been obtained from Wind database, CCER database, National Bureau of Statistics of China, and *China Statistical Yearbook*.

### 3.2 Variable definition and research design

**(a) Explained variable: Corporate financial performance, including return on total assets and corporate market value.** This study adopts the relative index generally used in academia to measure the corporate value, i.e., ROA. The method of calculation was the ratio of the current net profit to the average total assets. Further, this study adopted the relative index generally used in academic circles to measure enterprise value, i.e., tobinsQ, and the method of calculation was the ratio of market value to total assets.
**(b) Explanatory variable: Implementation of the carbon emission trading policy.** According to the standardized DID regression method, there were three explanatory variables in this study: the time of implementation of carbon emission trading, and the status of an enterprise with respect to tread and its time treatment. The implementation time and inclusion of carbon emission trading were dummy variables.
**(c) Moderating variables.** According to the relevant literature, the legal environment data have been obtained from the market intermediary organization development and the rule of law environment index in the China Province Market Index Report. According to the annual median, areas with higher than median have been classified having better legal environment, whereas those with lower than median were classified having worse legal environment.
**(d) Control variables.** The influence of the carbon emission trading on the enterprise value has also been affected by other factors. The control variables that are frequently used in the previous literature have been added to the model. According to the studies of Luo and Tang [38,43], six control variables have been selected to control the influence of other factors on the enterprise value. The control variables were current liquidity ratio, firm size, debt ratio, total asset turnover, equity concentration, and investor sentiment. The definitions of the main variables involved in the empirical test are listed in Table 1 below.

**Table 1:** Descriptions of variables.

Variables	Notation	Definition
Return on assets	Roa(roa)	Net profit/net assets
Firm market value	TobinsQ (tobinsQ)	Market value/ total assets
Time of implementation	Time (time)	The time of carbon emissions trading implementation
Scope of implementation	Treated (treated)	Enterprises are included in carbon emission trading. “Yes” or “No”
Legal environment	Law (law)	The legal environment, strict regulation is 1, loose regulation is 0
Current ratio	Current (current)	Current liquidity ratio
Firm size	Size (size)	The natural logarithm of total assets of a company at the end of a year
Debt ratio	Leverage (lev)	Total debt/Total assets
Total asset turnover	Turnover(turnover)	Current turnover of total assets
Equity concentration	Shrhfd	The Herfindahl index of top five shareholders
Investor sentiment	Investor (investor)	Mean of the investor sentiment index

### 3.3 Model construction

Based on the studies [44–49], the models (1)–(2) have been constructed and the OLS method has been employed for the regression analysis. The specific model is given as follows.


$$roa_{i,t}=\alpha_{0}+\alpha_{1}time+\alpha_{2}treated+\alpha_{3}time\times treated+\gamma x_{i,t}+\varepsilon_{i,t}$$ (1)

$$tobinsQ_{i,t}=\alpha_{0}+\alpha_{1}time+\alpha_{2}treated+\alpha_{3}time\times treated+\gamma x_{i,t}+\varepsilon_{i,t}$$ (2)

The roa_i,t_ represents the financial performance of the enterprise, which is measured by the return on total assets. The term tobinsQ_i,t_ represents the current enterprise value, which is measured by Tobin’s Q value. The term *a*_0_ is the intercept and *a*_*i*_ is the coefficient. The variable treated indicates whether the company has been treated in a carbon trading system, and time*treated is the cross-product of time and treated. The X_i,t_ represents the control variables, which includes current ratio (current), firm size, debt ratio (lev), total asset turnover (turnover), equity concentration (Shrhfd), and investor sentiment (Investor). Furthermore, ε_i_ represents the residuals.

## 4. Results and analysis

### 4.1 Descriptive statistics

Table 2 lists the descriptive statistics of the main variables (absolute value data adopts Winsor, standardized processing). The value of Chinese companies (tobinsQ) ranged from 0.151 to 12.99, reflecting the significant differences over the value among companies. Financial performance (Roa) also reflected the profitability gap among different companies. Both the explanatory variables, whether the carbon emission trading is implemented (time) and whether companies are included in carbon emission trading (treated), were dummy variables, with the mean values of 0.524 and 0.0434, respectively. The implementation scope mainly targeted the enterprises which were heavy polluters.

**Table 2:** Descriptive statistics.

Variables	Sample size	Mean	Median	Std. Dev.	Min	Max
tobinsQ	21592	2.401	1.752	2.231	0.151	12.99
Roa	21592	0.0396	0.0334	0.0441	-0.0504	0.133
time	21592	0.524	1	0.499	0	1
treated	21592	0.0434	0	0.204	0	1
time*treated	21592	0.0218	0	0.146	0	1
current	21592	3.505	1.609	84.35	-5.132	12223
size	21592	0.000839	-0.0739	1.020	0.0783	37.49
lev	21592	0.444	0.421	0.557	0.195	63.97
turnover	21581	0.619	0.508	0.534	0.000434	11.42
Shrhfd	21592	0.148	0.110	0.124	0.0000008	0.810
Investor	21592	54.82	55.02	4.661	28.17	73.59
Law	12782	0.4683	0	0.499	0	1

### 4.2 Main regression results

Table 3 shows the regression relationships between the carbon emission trading and corporate value, and the financial performance and R&D investment. Result (1) was employed to test Hypothesis 1, result (2) to test Hypothesis 2, and results (3)–(6) to show the regressions, in the case of grouping by law, to verify Hypothesis 3. The explained variables in each model were Roa and tobinsQ, with current ratio, enterprise size, asset-liability ratio, turnover capacity, equity concentration, and investor sentiment as control variables.

**Table 3:** The regression results.

Variables	(1)	(2)	(3)High law	(4)Low law	(5)High law	(6)Low law
	Roa	tobinsQ	Roa	Roa	tobinsQ	tobinsQ
time	-0.005***	-0.397***	-0.009***	0.003	0.609***	1.263***
	(-3.86)	(-5.92)	(-3.53)	(1.22)	(5.52)	(11.01)
treated	-0.006***	-0.211**	-0.009	-0.002	-0.171	0.051
	(-2.77)	(-2.25)	(-1.52)	(-0.88)	(-0.69)	(0.41)
time*treated	0.005*	-0.380***	0.022**	-0.002	-0.157	-0.396**
	(1.77)	(-2.93)	(2.50)	(-0.50)	(-0.41)	(-2.36)
current	0.000	0.000	-0.000	0.000***	-0.000	0.019***
	(0.16)	(0.34)	(-1.53)	(2.95)	(-0.51)	(6.99)
size	-0.001***	-0.008	0.046**	-0.014***	-9.604***	-1.176***
	(-3.39)	(-0.46)	(2.45)	(-3.93)	(-11.62)	(-7.44)
lev	-0.010***	-0.106***	-0.083***	-0.020***	-2.875***	-0.460***
	(-19.81)	(-4.32)	(-32.04)	(-13.65)	(-25.30)	(-6.86)
Turnover	0.007***	-0.011	0.013***	0.007***	-0.046	-0.050
	(12.13)	(-0.38)	(10.28)	(7.57)	(-0.84)	(-1.13)
Shrhfd	0.020***	-0.346***	0.023***	0.021***	0.088	-0.237
	(8.16)	(-3.03)	(5.32)	(4.91)	(0.46)	(-1.23)
Investor	0.002***	-0.056***	0.002***	0.002***	-0.039***	-0.017***
	(30.75)	(-18.74)	(13.81)	(13.21)	(-7.16)	(-3.17)
Constant	-0.076***	5.903***	-0.031***	-0.066***	5.339***	2.965***
	(-17.98)	(23.79)	(-3.58)	(-5.47)	(14.18)	(5.51)
Year control	Yes	Yes	Yes	Yes	Yes	Yes
Industry control	Yes	Yes	Yes	Yes	Yes	Yes
Observations	21592	21592	5,596	6,396	5,596	6,396
R-squared	0.104	0.245	0.252	0.112	0.302	0.267
Adj_R2	0.102	0.241	0.247	0.108	0.298	0.263
F	73.27	71	60.32	24.39	77.75	70.08

The t-statistics in parentheses (***p<0.01, **p<0.05, *p<0.1).

It can be seen from Table 3 that models (1) and (2) tested the fundamental hypothesis of this paper, besides the impact of the implementation of the carbon emission trading scheme on the total ROA of the enterprise and its corporate value in the capital market. The regression results showed that with the adoption of the DID method, the variable time*treated in result (1) had a significantly positive correlation with the Roa of the company at the 10% level. This indicated that, subsequent to the implementation of the carbon emission trading scheme, the financial performance of the involved enterprises was significantly improved, and H1 was verified. The time*treated in Result (2) had a significantly negative correlation with the corporate value in the capital market at the 1% level, indicating that, subsequent to the implementation of the carbon emission trading scheme, the values of enterprises that were included in carbon emission trading, compared with those not included, were significantly reduced, and H2b was verified.

In results *4*.*3* and *4*.*4*, time*treated in the strict legal environment group had a significantly positive correlation with Roa at the 5% level, whereas the correlation was not significant in the loose legal environment group. This proved that in the regions with more complete laws, enterprises participating in carbon emission trading had achieved high ROAs. Therefore, H3a has been verified. In results (5) and (6), time*treated had a significantly negative correlation with tobinsQ at the 5% level in the loose legal environment group, despite the correlation being not significant in the strict legal environment group. This proved that in the regions where the government supervision was weak, the capital market played a stronger role, and H3b was verified. Among the control variables, the leverage ratio had a significantly negative correlation with Roa and tobinsQ at the level of 1%.

### 4.3 Relieving the endogenous

Because the carbon emission trading is still in the pilot stage in China, its trading volume is smaller than the overall trading volume of the capital market, and hence only a few listed companies are included (78 enterprises in 2014, and the number has been increasing in the following years, reaching 137 in 2018). There may be deviations in the regression analysis with the full sample, hence, the estimation result heavily depends on the selection of the control group. For the sake of rigid measurement, this paper adopted the test method of propensity score matching and difference in differences (PSM-DID) to alleviate the endogenous effects. The treatment group referred to enterprises which had implemented the carbon emission, and the propensity score matching method was used to further match the control group in companies, which did not implement the carbon emission.

Specifically, the binary probity model was employed to estimate the probability that the sample was a company that implemented carbon emission control. Existing research shows factors that affect the carbon emission trading of an enterprise that includes the region, industry, scale, and profitability. Therefore, this work selected the size of an enterprise (size), the industry it was engaged in, the lev ratio (lev), the total operating income (income), the total operating cost (cost), and the area it was located in (area), as the matching variables in the prediction model. According to the propensity score, the group that was closest to the probability value of the treatment group was selected as the control group.

To ensure the accuracy of the results obtained with the PSM method, this study conducted a balance test, and the results are presented in Table 4. According to the table, the deviations of all variables after the matching were significantly reduced, all within 10%. Furthermore, all the post-matching p values of the *t-*test were greater than 10%, indicating the absence of significant differences in the post-matching between all variables of the treatment group and those of the control group. The descriptive statistics after PSM processing (omitted due to limited space, but available upon request) revealed that, in terms of the explained variables, the value of the Chinese companies varied from the minimum 0.0454 to the maximum 121.5 (non-standardized original value), showing that the differences between the enterprise values were obviously reduced, and the same was true for financial performance (Roa). In terms of the explanatory variables, the mean value of the implementation time of the carbon emission trading remained stable, and the mean value of the treated variables of companies included in the carbon emission trading increased to 0.342, indicating that the results met the processing requirements of PMS.

**Table 4:** PSM balance test results.

Variables	Mean value				Deviation reduction ratio(%)	*t*-test	
	Sample matched	Treatment group	The control group	Deviation rate(%)		*t*-value	p>|t|
Size	No	1.1312	-0.02866	29.4	97.4	14.99	0.000
	Yes	0.14206	0.11146	0.8		0.30	0.761
Ind	No	4.0927	4.9601	-28.2	83.8	-3.72	0.000
	Yes	3.8958	3.7556	4.6		0.72	0.474
Lev	No	0.48907	0.4159	32.8	86.4	5.23	0.000
	Yes	0.47462	0.46468	4.5		0.49	0.625
Income	No	0.38151	-0.037	34.3	73.6	11.61	0.000
	Yes	0.16554	.05516	9.0		1.30	0.195
Cost	No	0.33301	-0.013	27.9	61.7	5.34	0.000
	Yes	0.18967	.05728	10.7		1.41	0.158
Area	No	144.49	251.1	-70.7	97.7	-10.19	0.000
	Yes	148.47	146.04	1.6		0.20	0.845

After the PSM processing, this paper has re-examined the above model, and the results are presented in Table 5 below. The results showed that the regression results of time*treated on tobinsQ were basically consistent with the above one, and the significance of time*treated on Roa that improved and in a loose legal environment showed a negative correlation at the 10% level, further verifying that H3a was on another dimension. The above results proved that the conclusion of this paper was relatively stable with no significant endogenous problem.

**Table 5:** The regression results after the PSM balance test.

Variables	(1)	(2)	(3)High law	(4)Low law	(5)High law	(6)Low law
	Roa	tobinsQ	Roa	Roa	tobinsQ	tobinsQ
Time	-0.013***	-0.597***	-0.004	0.013**	-0.444	1.198***
	(-3.50)	(-3.51)	(-0.37)	(1.98)	(-1.01)	(4.25)
treated	-0.010***	-0.360***	-0.008	0.002	-0.359	-0.022
	(-4.17)	(-3.22)	(-1.14)	(0.47)	(-1.37)	(-0.14)
time*treated	0.010***	-0.312**	0.019*	-0.009*	**-0.045**	-0.482**
	**(3.03)**	**(-2.08)**	**(1.88)**	**(-1.94)**	**(-0.11)**	**(-2.33)**
current	-0.000	0.008**	-0.001**	-0.000	-0.022	-0.006
	(-0.94)	(2.05)	(-2.09)	(-0.48)	(-0.91)	(-0.89)
size	-0.000	-0.179**	-0.134	0.007	-15.102***	-0.879*
	(-0.12)	(-2.29)	(-1.49)	(0.62)	(-4.50)	(-1.81)
lev	-0.016***	-0.415***	-0.093***	-0.077***	-2.269***	-3.615***
	(-7.17)	(-3.99)	(-7.90)	(-10.46)	(-5.14)	(-11.48)
Turnover	0.015***	0.302***	-0.004	0.014***	-0.015	0.207*
	(7.77)	(3.47)	(-0.56)	(4.96)	(-0.06)	(1.69)
Shrhfd	0.033***	-0.392	0.037*	0.012	0.004	-0.523
	(5.10)	(-1.31)	(1.76)	(1.21)	(0.00)	(-1.22)
Investor	0.002***	-0.038***	0.001*	0.002***	0.006	-0.037***
	(9.05)	(-4.37)	(1.73)	(5.72)	(0.27)	(-2.74)
Constant	-0.068***	4.457***	0.014*	-0.051*	2.105*	5.520***
	(-4.93)	(7.12)	(1.37)	(-1.94)	(1.50)	(4.94)
Year control	Yes	Yes	Yes	Yes	Yes	Yes
Industry control	Yes	Yes	Yes	Yes	Yes	Yes
Observations	2,666	2,666	1290	1,120	1290	1,120
R-squared	0.214	0.305	0.337	0.189	0.351	0.346
Adj_R2	0.189	0.283	0.272	0.169	0.286	0.329
F	8.481	13.68	5.149	9.422	5.462	21.36

The *t*-statistics in parentheses.

***p<0.01,

**p<0.05,

*p<0.1.

### 4.4 Further analysis

#### 4.4.1 Impact of the implementation of carbon emission rights on enterprises’ R&D investment

According to the Porter Hypothesis, besides their impact on financial performance of the enterprises, various types of environmental regulations have more important impact on their R&D investment. It holds that technology is the key to protecting the environment and promoting economic growth. Recently, there are a significant amount of literature on numerous theoretical and practical research on the interaction between the environmental regulation and technology, which mostly revolves around the relationship between environmental regulation and technological, patent output, and R&D input. Schelling [50] and Lanjouw et al. [51] verified the relationship between the invention and diffusion of environmental technology and pollution control expenditures. Furthermore, they proposed that with the increase in pollution control expenditures, the number of environmental patents increased accordingly, i.e., strengthening environmental regulation could promote the technological innovation of the enterprises. Jaffe [52] found that the environmental regulation promoted technological investments of US manufacturing companies. For every 0.15% increase in pollution control expenditures, there was a 1% increase in green R&D investment. Recent studies by D’Agostino [41] and Goodchild et al. [22] have also supported the view above. However, as the carbon emission trading market of China is still in the pilot and developmental stage, several scholars have not yet reached a consensus on the role of carbon emission trading in promoting R&D investment.

Based on the above research, the impact of the carbon emission trading system on corporate innovation behavior needs to be analyzed by taking into account the situation of an enterprise before and after the implementation of this policy. It cannot be asserted that, subsequent to the implementation of carbon emission trading, Chinese enterprises have increased their investment in R&D activities. Enterprises may have improved their production processes through R&D investment, which can save quotas for financing or selling, and can obtain greater expected returns. On the contrary, owing to the loose restrictions the current carbon trading market in China, enterprises may be inclined more to carry out emission reduction activities by purchasing clean energy or environmental protection equipment. Therefore, this work further set the R&D intensity of an enterprise as the explanatory variable. Likewise, to alleviate endogenous effects, a sample after the propensity matching score was used for testing. The settings of other variables remain unchanged. For specific indicators, see the variable definition table above. The test results are listed in Table 6.

**Table 6:** Further studies examining the regression results.

Variables	a.	b.	c.
	R&D	Non-operating	investment
Time	0.956***	0.289***	0.245***
	(4.57)	(8.75)	(7.97)
Treated	0.311	0.329***	0.040
	(1.28)	(7.16)	(0.93)
time*treated	-0.043	0.091*	0.076
	(-0.14)	(1.71)	(1.26)
Current	0.067***	-0.000	-0.000
	(11.70)	(-0.45)	(-0.35)
size	-1.034***	0.127***	0.040***
	(-2.93)	(17.54)	(5.93)
lev	-3.417***	0.167***	0.093***
	(-19.97)	(13.89)	(8.26)
Turnover	-2.078***	0.063***	-0.001
	(-28.21)	(4.64)	(-0.07)
Shrhfd	-2.352***	0.581***	0.662***
	(-8.77)	(10.53)	(12.75)
Investor	0.059***	0.020***	0.026***
	(7.89)	(13.87)	(19.13)
Constant	0.492	-1.461***	-1.957***
	(0.90)	(-14.52)	(-20.70)
Year control	Yes	Yes	Yes
Industry control	Yes	Yes	Yes
Observations	13,554	21,504	21,351
R-squared	0.333	0.101	0.211
Adj_R2	0.331	0.0997	0.210
F	198.2	71.04	167.5

The *t*-statistics in parentheses.

***p<0.01,

** p<0.05,

* p<0.1.

#### 4.4.2. The impact of the implementation of carbon emission rights on an enterprise’s non-operating income and investment income

Certain scholars have proposed that the enterprises can obtain energy-saving funds, trading income, and extra profits through cleaner production projects. From the perspective of the accounting treatment of actual carbon emission trading of enterprises, the current carbon emission trading market in China is still in the pilot stage, and the project surveys have found that most of the enterprises conduct accounting confirmation in a simplified manner. Currently, enterprises mainly use accounting measurement when the quota purchase or sale, and include all the economic items in the non-operating income or cost by using the historical cost method (mainly in high-emission manufacturing industries such as steel, cement, and metal). On the contrary, enterprises dominated by the power industry and carbon asset management and investment institutions usually adopt the measurement attributes of fair value, and the income from carbon emission trading and changes in fair value have been included in the investment income. Therefore, the most direct impact of carbon emission trading on the financial performance of current Chinese enterprises is reflected in their non-operating income, besides their investment income. Thus, we believe that the implementation of carbon emission trading can enhance the nonoperating income and investment income of an enterprise.

Based on the above analysis, this work further sets the current non-operating income and investment of an enterprise as explanatory variables, and also uses the sample before the testing of the propensity matching score. The settings of other variables remain unchanged. For specific indicators, see the variable definition in the table above. The test results are presented in Table 6.

The test results showed that the variable time*treated in Result a. was not positive, yet insignificantly correlated with the R&D input of an enterprise. Therefore, it cannot be concluded that subsequent to the implementation of the carbon emission trading mechanism, enterprises incorporated in the carbon emission trading will strengthen the intensity of their R&D investment. The explanatory variable time*treated in result b was significantly and positively correlated with the nonoperating income at the 10% level, proving that the implementation of carbon emission trading has indeed increased the level of nonoperating income of companies included in the trading system. However, the correlation between the explanatory variable and the ROI in Result c. was not significant, which could not prove that the implementation of the carbon emission trading improved ROIs of enterprises. This may owe to the fact that most of the enterprises currently record only transaction quotas, instead of making accounting recognition for free quotas, resulting in a small overall amount and hence an insufficient impact on their ROIs. The tests of Results b. and c. also verified the current accounting treatment of enterprises and provided data support for *the Interim Provisions on Accounting Treatment for Carbon Emission Trading* issued by the Ministry of Finance of China, which included enterprises with non-free transaction of carbon emission quotas in their non-operating income and non-operating cost.

#### 4.4.3. The impact of the State-owned enterprise on main regression results

Considering that China has a large number of state-owned enterprises, the nature of this property right may affect the relationship between carbon trading and corporate financial performance. Therefore, we further tested the results under different property rights. The results show that in the group of soe = 1 (The sample is state-owned enterprise), the results of time*treated to Roa and tobinsQ are not significant. In the group of soe = 0 (The sample is not state-owned enterprise), the results of time*treated to Roa and tobinsQ are significant. This proves that China’s state-owned enterprises participate in carbon emissions trading did not have a significant impact on financial performance and corporate value, but non-state-owned enterprises can improve their financial performance through carbon emissions trading and reduce corporate value. The test results are presented in Table 7.

**Table 7:** Further studies result.

Variables	a.	b.	c.	d.
	Roa Soe = 1	Roa Soe = 0	tobinsQ Soe = 1	tobinsQ Soe = 0
Time	-0.015***	0.002	0.095	-0.390***
	(-8.54)	(1.17)	(1.33)	(-3.70)
Treated	-0.004	-0.005*	-0.195*	0.064
	(-1.62)	(-1.91)	(-1.94)	(0.43)
time*treated	**0.004**	0.006*	**-0.089**	-0.608***
	**(1.21)**	**(1.87)**	**(-0.60)**	**(-3.09)**
Current	0.000	-0.000	0.002	0.000
	(0.04)	(-0.11)	(1.32)	(0.80)
size	-0.000	-0.001***	-0.022	-0.030
	(-0.64)	(-3.13)	(-1.24)	(-1.40)
lev	-0.050***	-0.005***	-1.167***	0.002
	(-31.21)	(-9.30)	(-17.99)	(0.07)
Turnover	0.009***	0.009***	0.010	-0.039
	(10.47)	(11.35)	(0.30)	(-1.00)
Shrhfd	0.015***	0.034***	-0.949***	0.411**
	(4.76)	(10.16)	(-7.17)	(2.43)
Investor	0.002***	0.002***	-0.040***	-0.053***
	(18.24)	(24.85)	(-10.32)	(-12.74)
Constant	-0.051***	-0.086***	5.311***	5.664***
	(-8.41)	(-14.62)	(21.39)	(19.17)
Year control	Yes	Yes	Yes	Yes
Industry control	Yes	Yes	Yes	Yes
Observations	8,332	13,249	8,332	13,249
R-squared	0.208	0.106	0.175	0.210
Adj_R2	0.205	0.103	0.172	0.208
F	66.05	45.84	53.27	103.6

The *t*-statistics in parentheses.

***p<0.01,

** p<0.05,

* p<0.1.

#### 4.5 Robustness test

To enhance the reliability of the empirical conclusions, this section conducts a robustness test.

**(a) We have made adjustments owing to the hysteresis of the explanatory variables.** To prevent the “lag effect” of the carbon emission trading on the financial performance and market value of an enterprise, a re-examination has been conducted by adopting the Roa and TobinsQ lag data. The samples processed by PSM have been selected and regression tests were conducted on models (1) and (2) respectively (the results are shown in Table 8 below).
**(b) Adjustments have been made for the explanatory variables in basic regression analysis.** ROA has been replaced with return on equity (ROE), and the R&D intensity has been replaced with the total amount of R&D investment. Further, the impact of the carbon emission trading on the financial performance and R&D investment of an enterprise has been re-examined. Two samples before and after PSM processing have been selected, and the regression tests have been conducted on models (1) and (2), respectively (the results are presented in Table 7).

**Table 8:** Robustness test results.

VARIABLES	(1)after PSM	(2)after PSM	(3)before PSM	(4)before PSM	(5)after PSM	(6)after PSM
	Roa.l	Tobinsq.l	roe	R&DSum	roe	R&DSum
time	-0.002	0.875***	-0.015***	0.141***	-0.018**	0.244
	(-0.45)	(4.63)	(-6.41)	(2.82)	(-2.51)	(0.67)
treated	-0.007***	-0.287**	-0.006	0.488***	-0.013***	0.449**
	(-2.77)	(-2.46)	(-1.58)	(7.86)	(-2.81)	(2.33)
time*treated	0.008**	-0.350**	0.007*	**-0.208**	0.008*	**-0.217**
	**(2.24)**	**(-2.23)**	**(1.41)**	**(-0.66)**	**(1.29)**	**(-0.91)**
current	-0.000	0.007*	0.000	0.001	-0.000	0.010
	(-1.00)	(1.92)	(0.04)	(1.27)	(-0.78)	(1.15)
size	-0.000	-0.203**	0.003***	3.423***	0.000	2.834***
	(-0.17)	(-2.48)	(4.75)	(43.51)	(0.01)	(10.29)
lev	-0.022***	-0.815***	-0.000	0.398***	-0.003	1.498***
	(-9.00)	(-7.66)	(-0.04)	(9.52)	(-0.54)	(4.94)
Turnover	0.011***	0.231**	0.019***	0.051***	0.030***	0.081
	(5.39)	(2.54)	(17.91)	(2.83)	(8.58)	(0.56)
Shrhfd	0.019***	-0.378	0.045***	0.296***	0.047***	0.521
	(2.73)	(-1.20)	(10.19)	(4.23)	(4.01)	(1.12)
Investor	0.002***	-0.056***	0.004***	0.016***	0.004***	0.035**
	(7.13)	(-6.03)	(31.07)	(8.08)	(10.10)	(2.50)
Constant	-0.057***	4.494***	-0.153***	-1.123***	-0.141***	-3.055***
	(-3.68)	(6.54)	(-19.84)	(-8.12)	(-5.90)	(-2.97)
Year control	Yes	Yes	Yes	Yes	Yes	Yes
Industry control	Yes	Yes	Yes	Yes	Yes	Yes
Observations	2,420	2,420	21,581	13,951	2,665	1,804
R-squared	0.198	0.323	0.100	0.170	0.136	0.100
Adj_R2	0.170	0.299	0.0986	0.168	0.126	0.0857
F	7.035	13.58	70.39	83.85	13.81	6.826

The empirical results showed that the implementation of the carbon emission trading can still have a significant positive impact on the ROE of an enterprise, but it did not have a significant impact on the sum of the R&D investment of the enterprise. The test results have not changed substantially, indicating that the conclusion of this study is relatively robust.

## 5. Conclusions and further development suggestions

### 5.1 Discussion and conclusions

With the development of the carbon emission pilot trading market in China, the implementation of carbon emission trading has a key impact on the production and operation decisions of Chinese enterprises. We have selected Chinese A-share listed companies from 2009 to 2018 as samples, and analyzed the impact of carbon emission trading on corporate financial performance by employing the DID method. Evidence shows that the implementation of carbon emission trading could effectively improve the total asset-liability ratio of enterprises. However, it will reduce the value of the current capital market. Moreover, in the regions with strict legal environment, the increasing effect of the total asset-liability ratio was more obvious, whereas in the regions with loose legal environment, the reduction effect of the value of the capital market was more obvious. Further analysis showed that the implementation of the carbon emission trading could not promote the Chinese enterprises to increase R&D investment. The implementation of carbon emission trading has improved the level of non-business income of enterprises incorporated into the trading system. However, its impact on the investment income of enterprises was not significant.

The results European Union and China has been compared with similar studies in the United States. The conclusions that we have obtained in a bias towards the “uncertainty hypothesis”. Currently, carbon emission trading in China has a certain financial promotion effect on enterprises, but it has not reached the ideal state of the Porter hypothesis. However, we believe that with the further development of carbon market in China, enterprises will be able to obtain corresponding benefits through the carbon emission control in the future.

### 5.2 Further development suggestions

Primarily, it needs to effectively design accounting standards for the carbon emission rights and promote the enterprises to actively participate in the carbon emission trading. The carbon emission trading also has the policy significance of environmental protection on the basis of trading, which is different from general economic matters. Therefore, the accounting standards for this economic concern need to ensure the reliability and feasibility of accounting, besides meeting the requirements of government regulations. During the establishment of carbon accounting standards, the trading products such as quota, CCER, and financial products should be classified, and the different asset accounts and accounting treatment methods should be established to avoid the simplification of the current trading income.

Second, it needs to distinguish the enterprise carbon information users, and enhance the role of carbon information to enhance the enterprise value. We suggest that the corporate carbon information users should be distinguished, such as the capital market and investors. The capital market and investors should pay increased attention to the economic effect of the carbon trading, and the trading information such as the emission reduction cost and gain of trading enterprises should be included in the disclosure. For the purpose of economic supervision, Chinese Securities Regulatory Commission requires enterprises to disclose the compliance information such as the accounting treatment methods and performance completion time. The NDRC and environmental protection authorities need to require the enterprises to disclose the environmental information such as the energy consumption, carbon emission, and emission reduction. Based on the basic information, the corresponding economic, compliance, and environmental information should be disclosed to enhance the role of carbon information in improving the enterprise value.

Third, the unified trading market in China, under construction, should conduct the carbon financial trading based on technology research and development to enhance the role of the carbon emission trading technology. The study showed that the current carbon emission trading is still in the stage of enterprise trading income and has no significant impact on the enterprise R&D and innovation. Therefore, we suggest that the unified trading market should be in the future design of carbon financial products for the research and development of energy saving, emission reduction, and environmental protection technologies. This is to promote the enterprises to strengthen the research and development innovation and enhance the role of carbon trading technology.

## Supporting information

S1 Data (XLSX)

## References

[1] Mo JL, Agnolucci P, Jiang MR, Fan Y. The impact of Chinese carbon emission trading scheme (ETS) on low carbon energy (LCE) investment. Energy Policy. 2016;89: 271–283. doi: 10.1016/j.enpol.2015.12.002

[2] Tu Q, Betz R, Mo J, Fan Y, Liu Y. Can carbon pricing support onshore wind power development in China? An assessment based on a large sample project dataset. J Clean Prod. 2018;198: 24–36. doi: 10.1016/j.jclepro.2018.06.292

[3] Mayer C, Breun P, Schultmann F. Considering risks in early stage investment planning for emission abatement technologies in large combustion plants. J Clean Prod. 2017;142: 133–144. doi: 10.1016/j.jclepro.2016.05.089

[4] Gao Y, Li M, Xue J, Liu Y. Evaluation of effectiveness of China’s carbon emissions trading scheme in carbon mitigation. Energy Econ. 2020;90. doi: 10.1016/j.eneco.2020.104872

[5] Kang JN, Wei YM, Liu L, Han R, Chen H, Li J, et al. The Prospects of Carbon Capture and Storage in China’s Power Sector under the 2°C Target: A Component-based Learning Curve Approach. Int J Greenh Gas Control. 2020;101: 103149. doi: 10.1016/j.ijggc.2020.103149

[6] Zhang X, Karplus VJ, Qi T, Zhang D, He J. Carbon emissions in China: How far can new efforts bend the curve? Energy Econ. 2016;54: 388–395. doi: 10.1016/j.eneco.2015.12.002

[7] Porter ME. America’s green strategy. Sci Am. 1991;264: 193–246. doi: 10.1038/scientificamerican0491-168

[8] Porter ME, Van Der Linde C. Toward a new conception of the environment-competitiveness relationship. J Econ Perspect. 1995;9: 97–118. doi: 10.1257/jep.9.4.97

[9] Dales JH. Land, Water, and Ownership. Can J Econ. 1968;1: 791. doi: 10.2307/133706

[10] Coase RH. The Problem of Social Cost. Classic Papers in Natural Resource Economics. London: Palgrave Macmillan UK; 1960. pp. 87–137.

[11] Dobos I. The effects of emission trading on production and inventories in the Arrow-Karlin model. International Journal of Production Economics. Elsevier; 2005. pp. 301–308. doi: 10.1016/j.ijpe.2004.06.028

[12] Hua G, Cheng TCE, Wang S. Managing carbon footprints in inventory management. Int J Prod Econ. 2011;132: 178–185. doi: 10.1016/j.ijpe.2011.03.024

[13] Hayek MN, Harwatt H, Ripple WJ, Mueller ND. The carbon opportunity cost of animal-sourced food production on land. Nat Sustain. 2020. doi: 10.1038/s41893-020-00603-4

[14] Janzen R, Davis M, Kumar A. Greenhouse gas emission abatement potential and associated costs of integrating renewable and low carbon energy technologies into the Canadian oil sands. J Clean Prod. 2020;272. doi: 10.1016/j.jclepro.2020.122820

[15] Becker V, Lager T, Schulz HD. Transfer functions simulating the coprecipitation of trace elements in unsaturated soils. Environ Geol. 2009;58: 1601–1609. doi: 10.1007/s00254-008-1666-5

[16] Zhang YJ, Liang T, Jin YL, Shen B. The impact of carbon trading on economic output and carbon emissions reduction in China’s industrial sectors. Appl Energy. 2020;260: 114290. doi: 10.1016/j.apenergy.2019.114290

[17] Wen F, Wu N, Gong X. China’s carbon emissions trading and stock returns. Energy Econ. 2020;86. doi: 10.1016/j.eneco.2019.104627

[18] Jaraite-Kažukauske J, Di Maria C. Did the EU ETS Make a Difference? An Empirical Assessment Using Lithuanian Firm-Level Data. Energy J. 2016;37. doi: 10.5547/01956574.37.2.jjar

[19] Yang L, Li F, Zhang X. Chinese companies’ awareness and perceptions of the Emissions Trading Scheme (ETS): Evidence from a national survey in China. Energy Policy. 2016;98: 254–265. doi: 10.1016/j.enpol.2016.08.039

[20] Zhang YJ, Peng YL, Ma CQ, Shen B. Can environmental innovation facilitate carbon emissions reduction? Evidence from China. Energy Policy. 2017;100: 18–28. doi: 10.1016/j.enpol.2016.10.005

[21] Li W, Zhang YW, Lu C. The impact on electric power industry under the implementation of national carbon trading market in China: A dynamic CGE analysis. J Clean Prod. 2018;200: 511–523. doi: 10.1016/j.jclepro.2018.07.325

[22] Goodchild A, Toy J. Delivery by drone: An evaluation of unmanned aerial vehicle technology in reducing CO2 emissions in the delivery service industry. Transp Res Part D Transp Environ. 2018;61: 58–67. doi: 10.1016/j.trd.2017.02.017

[23] Zhang C, Wang Q, Shi D, Li P, Cai W. Scenario-based potential effects of carbon trading in China: An integrated approach. Appl Energy. 2016;182: 177–190. doi: 10.1016/j.apenergy.2016.08.133

[24] Wang H, Chen Z, Wu X, Nie X. Can a carbon trading system promote the transformation of a low-carbon economy under the framework of the porter hypothesis?—Empirical analysis based on the PSM-DID method. Energy Policy. 2019;129: 930–938. doi: 10.1016/j.enpol.2019.03.007

[25] Pang R, Zheng D, Shi M, Zhang X. Pollute first, control later? Exploring the economic threshold of effective environmental regulation in China’s context. J Environ Manage. 2019;248. doi: 10.1016/j.jenvman.2019.109275 31466176

[26] Zhou B, Zhang C, Song H, Wang Q. How does emission trading reduce China’s carbon intensity? An exploration using a decomposition and difference-in-differences approach. Sci Total Environ. 2019;676: 514–523. doi: 10.1016/j.scitotenv.2019.04.303 31054412

[27] Han R, Yu BY, Tang BJ, Liao H, Wei YM. Carbon emissions quotas in the Chinese road transport sector: A carbon trading perspective. Energy Policy. 2017;106: 298–309. doi: 10.1016/j.enpol.2017.03.071

[28] Chen H, Hou H, Hu H, Shang Z, Zhu Y, Cai H, et al. Aeration of different irrigation levels affects net global warming potential and carbon footprint for greenhouse tomato systems. Sci Hortic (Amsterdam). 2018;242: 10–19. doi: 10.1016/j.scienta.2018.07.021

[29] Pérez J, de Andrés JM, Lumbreras J, Rodríguez E. Evaluating carbon footprint of municipal solid waste treatment: Methodological proposal and application to a case study. J Clean Prod. 2018;205: 419–431. doi: 10.1016/j.jclepro.2018.09.103

[30] Dutta A, Bouri E, Noor MH. Return and volatility linkages between CO2 emission and clean energy stock prices. Energy. 2018;164: 803–810. doi: 10.1016/j.energy.2018.09.055

[31] Yi L, Bai N, Yang L, Li Z, Wang F. Evaluation on the effectiveness of China’s pilot carbon market policy. J Clean Prod. 2020;246. doi: 10.1016/j.jclepro.2019.118954 32051670 PMC6961971

[32] Dhar S, Pathak M, Shukla PR. Electric vehicles and India’s low carbon passenger transport: a long-term co-benefits assessment. J Clean Prod. 2017;146: 139–148. doi: 10.1016/j.jclepro.2016.05.111

[33] Zubi G, Fracastoro GV, Lujano-Rojas JM, El Bakari K, Andrews D. The unlocked potential of solar home systems; an effective way to overcome domestic energy poverty in developing regions. Renew Energy. 2019;132: 1425–1435. doi: 10.1016/j.renene.2018.08.093

[34] Descheemaeker K, Oosting SJ, Homann-Kee Tui S, Masikati P, Falconnier GN, Giller KE. Climate change adaptation and mitigation in smallholder crop–livestock systems in sub-Saharan Africa: a call for integrated impact assessments. Reg Environ Chang. 2016;16: 2331–2343. doi: 10.1007/s10113-016-0957-8

[35] Bui B, Moses O, Houqe MN. Carbon Disclosure, Emission Intensity and Cost of Equity Capital: Multi-Country Evidence. SSRN Electron J. 2019. doi: 10.2139/ssrn.3318011

[36] Velte P, Stawinoga M, Lueg R. Carbon performance and disclosure: A systematic review of governance-related determinants and financial consequences. Journal of Cleaner Production. Elsevier Ltd; 2020. doi: 10.1016/j.jclepro.2020.120063

[37] Fontana S, D’Amico E, Coluccia D, Solimene S. Does environmental performance affect companies’ environmental disclosure? Harri Laihonen P, editor. Meas Bus Excell. 2015;19: 42–57. doi: 10.1108/MBE-04-2015-0019

[38] Luo L, Tang Q. Determinants of the Quality of Corporate Carbon Management Systems: An International Study. Int J Account. 2016;51: 275–305. doi: 10.1016/j.intacc.2016.04.007

[39] Brouwers R, Schoubben F, Van Hulle C, Van Uytbergen S. The initial impact of EU ETS verification events on stock prices. Energy Policy. 2016;94: 138–149. doi: 10.1016/j.enpol.2016.04.006

[40] Akeiber H, Nejat P, Majid MZA, Wahid MA, Jomehzadeh F, Zeynali Famileh I, et al. A review on phase change material (PCM) for sustainable passive cooling in building envelopes. Renewable and Sustainable Energy Reviews. Elsevier Ltd; 2016. pp. 1470–1497. doi: 10.1016/j.rser.2016.03.036

[41] D’Agostino D, Parker D. A framework for the cost-optimal design of nearly zero energy buildings (NZEBs) in representative climates across Europe. Energy. 2018;149: 814–829. doi: 10.1016/j.energy.2018.02.020

[42] Lin B, Ge J. Carbon sinks and output of China’s forestry sector: An ecological economic development perspective. Sci Total Environ. 2019;655: 1169–1180. doi: 10.1016/j.scitotenv.2018.11.219 30577110

[43] Luo L. The influence of institutional contexts on the relationship between voluntary carbon disclosure and carbon emission performance. Account Financ. 2019;59: 1235–1264. doi: 10.1111/acfi.12267

[44] Venmans F. Capital market response to emission allowance prices: a multivariate GARCH approach. Environ Econ Policy Stud. 2015;17: 577–620. doi: 10.1007/s10018-015-0105-6

[45] Martin R, Muûls M, Wagner UJ. The impact of the European Union emissions trading scheme on regulated firms: What is the evidence after ten years? Rev Environ Econ Policy. 2016;10: 129–148. doi: 10.1093/reep/rev016

[46] Ramirez-Contreras NE, Munar-Florez DA, Garcia-Nuñez JA, Mosquera-Montoya M, Faaij APC. The GHG emissions and economic performance of the Colombian palm oil sector; current status and long-term perspectives. J Clean Prod. 2020;258. doi: 10.1016/j.jclepro.2020.120757

[47] Naranjo Tuesta Y, Crespo Soler C, Ripoll Feliu V. Carbon management accounting and financial performance: Evidence from the European Union emission trading system. Bus Strateg Environ. 2021;30: 1270–1282. doi: 10.1002/bse.2683

[48] Zhang S, Jiang K, Wang L, Bongers G, Hu G, Li J. Do the performance and efficiency of China’s carbon emission trading market change over time? Environ Sci Pollut Res. 2020;27: 33140–33160. doi: 10.1007/s11356-020-09168-3 32529608

[49] Guan D, Shan Y, Liu Z, He K. Performance Assessment and Outlook of China’s Emission-Trading Scheme. Engineering. 2016;2: 398–401. doi: 10.1016/J.ENG.2016.04.016

[50] Schelling TC. Intergenerational discounting. Energy Policy. 1995;23: 395–401. doi: 10.1016/0301-4215(95)90164-3

[51] Lanjouw JO, Mody A. Innovation and the international diffusion of environmentally responsive technology 1. Res Policy. 1996;25: 549–571. doi: 10.1016/0048-7333(95)00853-5

[52] Jaffe RB. Is the academic physician-scientist an oxymoron in contemporary obstetrics and gynecology? Am J Obstet Gynecol. 1997;177: 892–893. doi: 10.1016/s0002-9378(97)70290-4 9369841

